# Interplay effects in highly modulated stereotactic body radiation therapy lung cases treated with volumetric modulated arc therapy

**DOI:** 10.1002/acm2.13028

**Published:** 2020-10-26

**Authors:** Desmond J. Fernandez, Justin T. Sick, Jonas D. Fontenot

**Affiliations:** ^1^ Department of Physics and Astronomy Louisiana State University Baton Rouge LA USA; ^2^ Mary Bird Perkins Cancer Center Baton Rouge LA USA

**Keywords:** 4D‐CT, interplay, MLC, SBRT, VMAT

## Abstract

Interplay effects in highly modulated stereotactic body radiation therapy lung cases treated with volumetric modulated arc therapy.

**Purpose:**

To evaluate the influence of tumor motion on dose delivery in highly modulated stereotactic body radiotherapy (SBRT) of lung cancer using volumetric modulated arc therapy (VMAT).

**Methods:**

4D‐CT imaging data of the quasar respiratory phantom were acquired, using a GE Lightspeed 16‐slice CT scanner, while the phantom reproduced patient specific respiratory traces. Flattening filter‐free (FFF) dual‐arc VMAT treatment plans were created on the acquired images in Pinnacle^3^ treatment planning system. Each plan was generated with varying levels of complexity characterized by the modulation complexity score. Static and dynamic measurements were delivered to GafChromic EBT3 film inside the respiratory phantom using an Elekta Versa HD linear accelerator. The treatment prescription was 10 Gy per fraction for 5 fractions. Comparisons of the planned and delivered dose distribution were performed using Radiological Imaging Technology (RIT) software.

**Results:**

For the motion amplitudes and periods studied, the interplay effect is insignificant to the GTV coverage. The mean dose deviations between the planned and delivered dose distribution never went below −2.00% and a minimum dose difference of −5.05% was observed for a single fraction. However for amplitude of 2 cm, the dose error could be as large as 20.00% near the edges of the PTV at increased levels of complexity. Additionally, the modulation complexity score showed an ability to provide information related to dose delivery. A correlation value (R) of 0.65 was observed between the complexity score and the gamma passing rate for GTV coverage.

**Conclusions:**

As expected, respiratory motion effects are most evident for large amplitude respirations, complex fields, and small field margins. However, under all tested conditions target coverage was maintained.

## 1 Introduction

Lung cancer is the second most diagnosed cancer in men and women, but the leading cause of cancer death among both.^1^ Lung cancer accounts for approximately 37% of all cancer mortality and the stage of the disease at diagnosis is heavily related to outcome. When an individual is diagnosed after the cancer has metastasized, the expected 5‐year survival is approximately 4%. In contrast, when an individual is diagnosed in the localized stage of the disease the expected 5‐year survival is approximately 54%. Although the number of individuals with a localized diagnosis represents a small percentage of the total number of diagnosed lung cancers, this number is expected to increase with the use of computed tomography (CT) scans as a lung cancer screening tool for high‐risk individuals. For localized lung cancers, surgical resection is currently considered the standard of care. However, many patients diagnosed with lung cancer are elderly or have health deficits that make them unsuitable candidates for surgery. Radiation therapy is the preferred treatment option for patients unwilling or unsuitable for surgery.^2^

Volumetric Modulated Arc Therapy (VMAT) is an advanced radiation delivery technique that delivers fluence modulated radiation fields while the gantry of a linear accelerator is rotating through one or more arcs. During arc delivery, near continuous variations of gantry rotation speed, dose rate, and multi leaf collimator (MLC) position modulate the beam's fluence in an effort to deliver a conformal dose distribution. When using fluence modulated techniques there are concerns of interplay between moving tumors and dynamically changing parameters, most notably MLC motion. Interplay is the result of treatment planning dose calculations on stationary CT data sets. A treatment volume, assumed to be stationary in treatment planning, is modulated by segments of time‐dependent changes in MLC and gantry positions. During delivery, the displacement of this volume, relative to the moving MLC leaves, may result in deviations between the planned and delivered dose distributions.

Previous investigations of interplay have been reported for lung cases using conventional fractionation schemes (eg 30 fractions at 2 Gy per fraction).^3^, ^4^, ^5^, ^6^, ^7^ Jiang et al used a motor driven platform to simulate one‐dimensional sinusoidal movements. Solid water was placed on top this platform with a Farmer chamber inside and clinical lung cancer treatment plans were delivered. Seco et al showed that for a sliding widow and step‐and‐shoot IMRT, up to an 18% dose variation was observed for a single fraction, under similar measurement conditions, observed interplay dose variations to be greatest for IMRT beam segments with low monitor units (MU). Chui et al, developed a computational algorithm that calculates the effects of respiratory‐induced organ motion on delivered dose. In this study, the effects of organ motion broadened the penumbra and degraded the coverage of the planning target volume for lung treatments were reported. Court et al, reported that dose variations were largest for small MLC separation and fast moving MLCs. Also, it was observed that dose variations were largest for complicated MLC sequences, large amplitudes, and single arcs. However, under clinical conditions, all studies above concluded that the use of multiple beams per fraction and several fractions per treatment course causes the interplay effect to be averaged out and dosimetrically insignificant for conventional fractionation schemes.

Stereotactic body radiation therapy (SBRT) is one specialized form of external beam radiation therapy treatment that utilizes hypofractionated radiation doses delivered in a limited number of fractions. Stereotactic treatments are characterized by small treatment volumes and sharp dose fall off into the surrounding healthy tissue. SBRT's hypofractionated doses have shown to be very effective at treating localized lung cancer.^8^ The small treatment volumes and sharp dose fall‐off limit the amount of healthy lung tissue treated; which is desirable considering that mean lung dose correlates with lung complications.^9^

Although multiple studies have investigated the effects of interplay on hypofractionated SBRT treatment regimes, the results are still inconclusive on both the overall impact of interplay and the main contributors to interplay effects. One study found significant changes in target coverage for highly modulated fields and large motion amplitude^10^ while another reported minimal interplay even as target excursion increased to 2–3 cm.^11^ A third study reported that interplay effects would be insignificant with sufficient margin.^12^ Another reported negligible interplay effects, although this study was limited by using minimal modulation in their treatment plans.^13^

At our clinic, SBRT treatments have been implemented since 2009. Over the course of the program, the treatment team has observed some circumstances of high plan modulation; such as when lung tumors are in close proximity to critical structures where significant dose sparing is required. Therefore, the goal of this study is to comprehensively assess lung VMAT SBRT dose delivery under increased levels of modulation and range of motion. Evaluation of target coverage was used to gauge interplay effects on the delivered dose distributions in an effort to evaluate its clinical importance. Gamma analysis was also used to assess the agreement between planned and delivered dose. Additionally, correlation of target coverage to a plan‐based modulation metric was used to provide an indication of potentially risky plans.

## 2 Methods

### 2.A Treatment planning

#### 2.A.1 Respiratory phantom

In this study, a dynamic respiratory phantom (Quasar Respiratory Phantom, Modus Medical Devices, Ontario, Canada) was used as a patient model. The phantom is composed of an acrylic body and an electric drive unit was designed to simulate one‐dimensional internal lung motion with various cylindrical “lung” inserts that move in the longitudinal direction while simultaneously simulating one‐dimensional external chest motion with a platform that moves in the anterior‐posterior direction. The phantom's acrylic body, internal and external motion features, and array of cylindrical inserts makes the device suitable for mimicking a breathing patient.

**Fig. 1:**
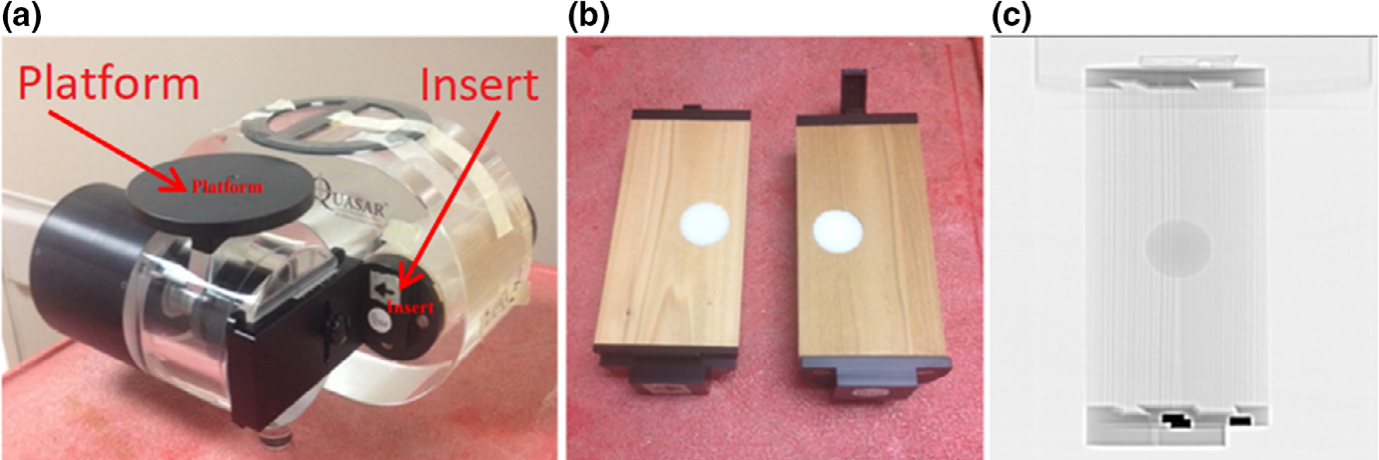
Quasar Phantom (a), Cedary lung tumor insert (b), CT scan of cedar lung tumor insert (c). The insert is composed of cedar wood and contains an offset, 3 cm diameter, plastic sphere to be used as lung and tumor surrogates respectively. The CT number and density of cedar ranges 290–400 and 0.25–0.32 g/cm^3^, which compares favorably with lung (140–300 and 0.15–0.33 g/cm^3^ respectively). The CT number and density of the plastic sphere was 950 and 0.98 g/cm^3^.

The cedar lung tumor insert was used for this study. The insert is composed of cedar wood and contains an offset, 3 cm diameter, plastic sphere to be used as lung and tumor surrogates respectively. The CT number and density of cedar ranges between 290–400 and 0.25–0.32 g/cm^3^, which compares favorably with lung (140–300 and 0.15–0.33 g/cm^3^ respectively^14^). The CT number and density of the plastic sphere was 950 and 0.98 g/cm^3^, which compares favorably with soft tissue (1000 and 1.02 g/cm^3^, respectively^14^). This insert, inside the phantom's acrylic body, provides the desired imaging conditions of an actual lung tumor patient. The phantom is shown in Fig. 1.

#### 2.A.2 Respiratory motion models

The control software for the phantom supports import of 1D respiratory traces that can be reproduced by the phantom. Using this feature, three anonymized patient‐specific breathing traces acquired with the external tracking system (Varian Real‐Time Positioning (RPM), Varian Medical Systems, Palo Alto, CA) were attained. Two traces were selected where the amplitude and period of the respiratory cycles were consistent. Additionally, one irregular trace was selected where the amplitude and period of the respiratory cycles varied. The provided software utilizes a waveform editor that allows the user to filter, compress, stretch, and scale the amplitude of a given waveform. The editing software was used to scale each waveform to fixed amplitudes of 1 and 2 cm, for a total of six traces.

#### 2.A.3 Treatment planning image acquisition

Currently, our clinic employs a motion encompassing imaging technique for managing intrafraction respiratory motion during treatment planning and delivery of lung SBRT. This technique involves creating a region of interest (ROI) that encompasses the target and its envelope of motion. Images of the phantom were acquired with a General Electric Lightspeed multi‐slice CT scanner (General Electric Company, Waukesha, WI). The parameters and values for the 4D‐CT gating protocol included slice thickness of 2.5 mm, 400 mAs, 30 cm field of view, 120 kVp, and variable (5–8 s) cine duration.

The imaging protocol consists of taking an initial scout scan, a free‐breathing (FB) CT scan and then a cine scan. During cine image‐acquisition, the Varian Real‐time Position Management (RPM) system, which consists of an infrared (IR) tracking camera and a reflective marker, monitors the external chest motion of the phantom and generates a breathing trace. Once acquired, the cine volumetric data and the RPM breathing trace were sent to the Advantage 4D‐CT v1.6 binning software (GE Healthcare, Buckinghamshire, England) to create respiration‐correlated CT datasets at four different breathing phases (eg full‐inhale, mid‐exhale, full‐exhale, mid‐inhale). Additionally, the Advantage software was used to create a maximum intensity projection (MIP) data set.

After imaging, the FB and MIP CT data sets were exported to a treatment planning system (TPS) (Pinnacle,^3^ Fitchburg, Wisconsin, U.S.). The FB and MIP were fused and the MIP was used to contour the ITV on the FB‐CT. A uniform margin of 0.5 mm was added to the ITV to form the PTV. Flattening filter free (FFF) VMAT plans were created according to the Radiation Therapy Oncology Group (RTOG) 0813 on the FB‐CT data sets. Key protocol requirements included 1) 60% < Normalization < 90%, 2) prescription isodose covers >95% of the PTV, 3) any dose >105% of Rx should occur in the PTV, and 4) 50% isodose line should be < 2 cm from PTV edge. Each FFF‐VMAT plan consisted of two full 360° treatment arcs at 6 megavoltage (MV) photon beam energy. The collimator angle was set to 45° and the couch angle was 0°. VMAT plans were generated using Pinnacle^3^ SmartArc inverse planning module.^15^ All plans were optimized using a 4° control points spacing and a 0.46 cm/degree leaf motion constraint.

#### 2.A.4 Complexity score

Plans were created at varying degrees of complexity characterized by the modulation complexity score (MCS). The MCS was utilized to quantify the MLC modulation of each plan. MCS was proposed by McNiven et al, for step‐and‐shoot IMRT^16^ and then adopted for VMAT^17^ as reported by Masi et al. The MCS has a fixed range from 0‐1, with lower scores indicating increased modulation. MCS is calculated based on three characteristics of each segment: shape, area, and weight.

Segment shape is quantified using the leaf sequence variability (LSV) parameter. LSV is defined as the variability in segment shapes of each field. The segment shape is based on the difference in leaf position between adjacent MLC leaves for each leaf bank excluding those positioned under the jaws. The maximum distance between positions for a leaf bank is defined as.

(1) $$pos_{\max}\left(\mathrm{CP}\right)=\max\left(pos_{n\in N}\right)‐\min{\left(pos_{n\in N}\right)}_{\mathrm{leafbank}}$$

The LSV is then calculated as follows:

(2) $${\text{LSV}}_{\mathrm{cp}}==\left(\frac{\sum_{n=1}^{N‐1}\left(pos_{\max}‐\left|\left(pos_{n}‐pos_{n+1}\right)\right|\right)}{\left(N‐1\right)xpos_{\max}}\right)x\left(\frac{\sum_{n=1}^{N‐1}\left(pos_{\max}‐\left|\left(pos_{n}‐pos_{n+1}\right)\right|\right)}{\left(N‐1\right)xpos_{\max}}\right)$$

Segment area is quantified using aperture area variability (AAV). AAV is defined as the variation in segment area relative to the maximum aperture area. Segments that is similar in area to the maximum aperture area contribute to a larger complexity score, ie less modulation. The AAV is calculated using the leaf position information as follows:

(3) $${\text{AAV}}_{\mathrm{cp}}=\left(\frac{\sum_{a=1}^{A}\left({〈pos_{a}〉}_{\mathrm{leftbank}}‐{〈pos_{a}〉}_{\mathrm{rightbank}}\right)}{\sum_{a‐1}^{A}\left({〈\max\left(pos_{a}\right)〉}_{leftbank\in arc}‐{〈\max\left(pos_{a}\right)〉}_{rightbank\in arc}\right)}\right)$$

where A is the number of leaves in the leaf bank.

Finally, the segment weight is incorporated into the complexity score. Control points with a larger number of MUs have a larger weighting and contribute more to the complexity score. The weighting is incorporated along with AAV and LSV into the final MCS calculation. The MCS for an entire arc, MCS_arc_, is the product of the LSV_cp_ and AAV_cp_ weighted by the relative MU of each control point in the beam.

MCS_arc_ is defined as follows:

(4) $${\text{MCS}}_{\mathrm{arc}}=\sum_{i=1}^{I‐1}\left[\frac{\left({\text{AAV}}_{\mathrm{cpi}}+{\text{AAV}}_{cpi+1}\right)}{2}x\frac{\left({\text{LSV}}_{\mathrm{cpi}}+{\text{LSV}}_{cpi+1}\right)}{2}x\frac{{\text{MU}}_{cpi,i+1}}{{\text{MU}}_{\mathrm{arc}}}\right]$$

The PTV prescription was set to 1000 cGy per fraction for 5 fractions and the dose was calculated on a dose grid of 3x3x3 mm^3^. This dose fractionation scheme was selected as it is allowed by the RTOG 0813 protocol, the current standard at our clinic for most cases, and common in the literature.^12^, ^13^, ^18^

Plans were created at varying degrees of complexity characterized by the modulation complexity score. This was achieved by contouring approximate regions of interest to represent normal anatomy. While the plastic insert inside the phantom represented a left lung tumor, regions of interest (ROI) approximating the location and size of the right lung, spinal cord, left ribs, and esophagus were delineated. Additionally, avoidance ROIs were contoured inside the ITV and PTV volume adjacent to the GTV to assist with generating greater MLC modulation in the more complex cases. Initial dose objective values were set according to the RTOG critical organ dose‐volume limit with an objective weight of one. The objective weight for each ROI value was progressively reduced until an objective value of 0.01 was reached. Critical structures were subsequently contoured in the phantom geometry to increase complexity and decrease the MCS in increments of 0.05. Successive treatment plans were created and the complexity score was steadily decreased until a MCS of 0.40 was reached, for a total of 45 treatment plans. Once the desired modulation complexity score was achieved, analysis of dose‐volume metrics, for agreement within protocol requirements, was performed. The plans and reference images were then exported from Pinnacle^3^ and imported into MOSAIQ Record and Verify System (Elekta AB, Mountainview, CA) information system for treatment delivery.

The calculated MCS for these plans resulted in a complexity score of 0.70 and 0.75 for the 1 cm and 2 cm target amplitude respectively. The MCS value of 0.40 was chosen as an end point since such values are comparable to those observed in head and neck IMRT treatment plans.^16^

### 2.B Measurements

#### 2.B.1 Film calibration

Linear accelerator output was measured before each film measurement session using the protocol recommended by Task Group 51 of the American Association of Physicists in Medicine.^19^ A Physikalisch‐Technische Werkstätten (PTW) GmbH farmer ionization chamber, SN: N30006‐0074, (PTW, Freiburg, Germany) was placed in a solid water phantom (CIRS, Norfolk, VA) and irradiated with 100 MU. The farmer chamber was coupled with a CNMC Model 206 dosimetry electrometer, SN: 11207335, (CNMC Company, Nashville, TN) in order to measure the charge collected per MU. The output measurements were converted to dose and film dose calibration curves were adjusted accordingly to account for changes in daily output.

Treatment plans were delivered to the respiratory phantom using an Elekta Versa HD linear accelerator (Elekta Oncology Systems, Crawley, UK). This accelerator has a 160‐leaf MLC supporting up to a 40 × 40 cm^2^ field size, resulting in a leaf width of 0.5 cm at isocenter. This accelerator is used clinically for SBRT procedures.

Two‐dimensional (2D) dose distributions of each treatment were measured using radiochromic film placed inside the cedar insert of the respiratory phantom. Radiochromic film was chosen because it is insensitive to ambient light, does not require wet chemical processing (ie self‐developing), and has high spatial accuracy. Insensitivity to ambient lighting greatly simplifies the processes of handling, cutting, and loading film. Self‐processing film eliminates any variations that may arise from wet chemical processing and high spatial accuracy is important when measuring dose distributions with steep dose gradients, such as those common in SBRT treatment. GafChromic EBT^3^ (International Specialty Products, Wayne, NJ), was the type of radiochromic film used to measure the dose distributions.

A calibration curve was prepared for each batch of film. In this process, two sheets of 8 × 10 in^2^ films were cut into twelve 3 × 3 in^2^ pieces and marked for orientation purposes. With one film piece not irradiated representing background, 11 pieces of film were exposed to a range of doses from 250 to 1425 cGy. Each calibration film was set perpendicular to the radiation delivery in solid water at a 100 cm SSD and 1.5 cm depth, d_max_ for 6 MV energy, with 10 cm of backscatter. Radiation was delivered with a 6 MV photon beam using an open 10 × 10 cm^2^ field. Per manufacturer recommendations, each film was stored for a period greater than 24 hr before scanning to ensure that all polymer changes (self‐development) had completed. Films were scanned in using an Epson Expression 10000XL flatbed scanner (Section 2.B.3) and a calibration curve was developed in Radiological Imaging Technology analysis software (Section 2.B.4) using a built in calibration procedure.

Before treatment delivery on the linear accelerator, a piece of radiochromic film was prepared by cutting the film flush with the cedar film and tumor insert and punching registration holes in each corner. The registration holes were punched using a hole puncher with consistent setting to punch a hole 7/16 and 1/2 inch from longitudinal and lateral edge of the film corner respectively.

The insert (now containing the film) was placed inside the phantom, which was aligned to the room lasers by radiopaque markers placed on the phantom during simulation. A kilovoltage cone beam computed tomography (kV‐CBCT) scan of the moving insert was taken using the x‐ray volume imaging (Elekta Versa HD, Elekta Ltd. Crawley, West Sussex, United Kingdom) system to align the phantom position with that in the planning CT data set exported from the TPS. Each kV‐CBCT was acquired with a bow‐tie filter (F1) and a small collimator field of view with an axial length of 26 cm (S20). Once the kV‐CBCT was acquired, it was aligned to the planning CT using automatic grey‐scale matching. The designated volume for registration (clipbox) was defined to encompass the stationary parts of the phantom. Treatment plans were then delivered to the phantom, and the resulting dose was measured, with and without respiratory motion.

#### 2.B.2 Planar dose export

The coronal dose plane corresponding to the dose distribution measured by the film inside the phantom was exported from the TPS for each treatment plan. Planar doses were calculated for a 20 × 20 cm^2^ square field. The planar dose tool, was used to create ASCII planar dose files at a resolution of 1 mm. The ASCII files were exported, and retrieved via file transfer protocol (FTP).

#### 2.B.3 Digitization of exposed films

Radiochromic film measurements were digitized using an Epson Expression 10000XL flatbed photo scanner (Seiko Epson Corporation, Nagano, Japan). This scanner was used to save 48‐bit red‐green‐blue (RGB), 150 dots per inch (DPI), images in tagged image file format (TIFF).

As recommended by the manufacturer, the films were scanned in landscape orientation to reduce lateral response artifacts. Care was taken to preserve film orientation and time between exposure and processing. Additionally, a cutout was designed to make sure that each film was placed in relatively the same position on the scanner during readout. Since the Epson scanner has no warm‐up process, though 10 repeated warm‐up scans were performed on the scanner before actual image digitization. Each film was digitized at 0.178 mm per pixel in order to balance resolution and document size.

#### 2.B.4 Film registration

Delivered and planned dose distributions were registered and analyzed in Radiological Imaging Technology (RIT) v6.3 analysis software (Radiological Imaging Technologies, Inc., CO). The Epson scanned 48‐bit RGB radiochromic film image was imported as a 16‐bit green channel image since fractionated doses were over 10 Gy. A 2D median filter of 5x5 pixels was applied to all imported film to reduce inherent image noise. The calibration curve, corresponding with the appropriate film batch, was applied to convert each film pixel value to dose. The planned planar dose ASCII file was also imported into RIT for image registration and analysis.

The planar dose image was registered to the film's measured dose distribution using a registration template created within the RIT software. The template was created by acquiring a CT scan of the phantom (stationary) with film inside and using the measurement tools in the TPS to determine the distances of the film registration holes from isocenter. Once the registration template was created and applied to the planar dose image, the film image and planar dose image were registered using a point‐based rigid body registration tool. The registered images were then normalized and analyzed.

### 2.C Uncertainty measurements

The treatment delivery and film analysis processes are subject to error. The quality of the results presented herein is directly related to this error. Measurements were performed to quantify the uncertainty in these steps.

The uncertainty in the CBCT software registration algorithm was determined by a process similar to that described in Sutton et al.^20^ The phantom was initially aligned to isocenter by a kV‐CBCT using grey‐value registration with only translation shifts. This gave the best alignment possible without considering rotations that the couch is unable to account for. After the phantom was aligned, six repeated measurements were acquired without moving the phantom by re‐calculating the registration. Ideally, these repeated measurements would have a mean and standard deviation of zero, with any deviations from that value indicating the inherent noise in the image guidance process. The mean ± standard error in the X, Y, and Z longitudinal directions, for N = 6 measurements, are 0.17 ± 0.24, −0.50 ± 0.10, and 0.48 ± 0.21 mm, with standard deviations of 0.59, 0.24, and 0.51 mm respectively.

The quality of the film and planar dose registration process was adopted from Vinci et al.^21^ The film registration software displays an estimated error value for each film registration point (δx_i_, δy_i_) by evaluating the geometric relationship between registration points in the planar dose and film dose images. These values were taken to directly quantify the quality of the registration process, Q, as calculated by:

$$Q=\sigma_{\mathrm{RIT}}=\left(\frac{1}{2N‐1}\right)\sum_{j=1}^{N}\left[{\left(\frac{\sum_{i=1}^{N}\left(\Delta x+\Delta y\right)}{2N}‐\Delta x_{j}\right)}^{2}+{\left(\frac{\sum_{i=1}^{N}\left(\Delta x+\Delta y\right)}{2N}‐\Delta y_{j}\right)}^{2}\right]$$

where N = the number of registration points.

The average, range and standard deviation of Q, for N = 30, was 0.60, 0.33 – 0.96, and 0.18 mm respectively. As a rule of thumb, the standard deviation ($\sigma_{\mathrm{RIT}}$) should be less than or equal to 1/Pmm, where Pmm is the pixel size of the reference image in mm. For the 1 mm pixel size of our reference images, the Q value from each registration should be less than 1 mm to be considered appropriate. This served as a quality check for all films.

Additionally, one film was registered 10 times to its corresponding planar dose distribution and evaluated. The deviation in quality (Q) from this process (σ_Q_ = 0.03 mm) was far less than the deviations observed from registering different films. This indicated that the error is in large part due to film preparation (*ie* the manual cutting of film to fit inside the insert and punching of film registration holes).

Lastly, one A/P plan was generated and delivered to the phantom three times in one session to measure the end‐to‐end variation in the phantom setup, treatment delivery, and film analysis. Each film delivery was registered to the corresponding planar dose file in RIT. Longitudinal and lateral profiles were acquired and the displacements between midpoints were determined using the 50% isodose line positions. This procedure includes all errors from kV‐CBCT alignment, from treatment delivery, and from film registration and scanning. As measured from the data, the displacements of the midpoint at the 50% dose level between films was 1.21 mm in the longitudinal direction and 0.17 mm in the lateral direction for the film deliveries.

### 2.D Analysis metrics

The various measurements and patient models examined in this work are summarized as follows: three breathing traces, seven complexities at 1 cm motion amplitude, and eight complexities at 2 cm amplitude, each plan was delivered five times. Analysis was performed on all static and dynamic film deliveries using the RIT V6.3 software package. Once the film and planned dose distributions were registered, five 1‐D profile measurements along the longitudinal (superior‐inferior) and lateral (right‐left) axis were acquired for each film. One profile through isocenter and four profiles directly adjacent to isocenter were acquired summed and averaged.

Target coverage was evaluated using the profile measurements. The analysis metrics evaluated the position of the measured dose distributions compared to the calculated distributions. The width of the 100% prescription dose, the width of the 95% prescription dose, and relative dose and percent dose error at the edges of the GTV, ITV, and PTV between planned and delivered dose were evaluated. Insufficient target coverage will be considered a relative dose below 95% of the prescription in the GTV and a relative dose below 90% of the prescription in the PTV. Additionally, the mean, minimum, and maximum dose difference for all points inside the GTV, ITV, and PTV were calculated between the planned and delivered distributions.

Additionally, the RIT software has the ability to perform gamma analysis. Gamma analysis^22^ was used to compare the agreement between planned and delivered distributions with the TPS calculation. Gamma analysis considers the dose difference and spatial displacement between each point. RIT's gamma analysis method was performed at gamma criteria of 3% dose difference and 3 mm distance to agreement. Gamma analysis was performed on the area representing the GTV (sphere), the ITV (MIP), and the PTV in the dose distribution. Since the exact position of isocenter is known on the pinnacle planar dose export, it was used to crop a region of interest that fully encompasses these areas. Additionally, the gamma passing rates were plotted against their corresponding MCS values.

## 3 Results

A summary of pertinent results can be found in Table 1.

**Table 1:** Summary of results.

Strong linear correlation (R = 0.92) between MCS and number of MU			
Range of dose differences at the longitudinal edges		Superior Edge	Inferior Edge
Dynamic Delivery @ 2 cm		[−4.06% −22.42%]	[6.96% −14.02%]
Dynamic Delivery @ 1 cm		No Trend	
Relative dose at the longitudinal edges (Rx = 1000 cGy)			
All amplitudes	GTV	1137 cGy–1366 cGy	
	ITV	1054 cGy–1263 cGy	
PTV @ 1 cm		934 cGy–1131 cGy	
PTV @ 2 cm		826cGy–1163 cGy	
Gamma Analysis			
Complexity ↑, Gamma Passing Rate ↓			
Dynamic < Static			
2 cm Motion Had Lowest Gamma Passing Rates			

### 3.A Plan monitor units

**Fig. 2:**
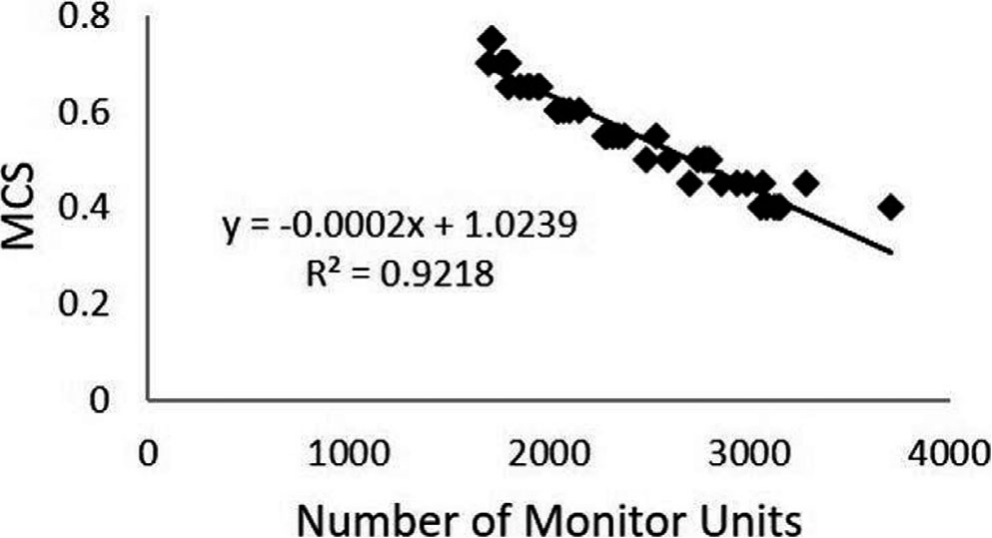
MCS vs MU. The relationship between the number of MU and the MCS is shown. The graph shows that there was a strong linear correlation (R = 0.92) between MCS and the number of MU.

The total number of MU was recorded. The number of MU in each plan generally increased with increasing plan modulation for all three patients. Figure 2 depicts the relationship between the number of MU and the MCS for each plan evaluated. The graph shows that there was a strong linear correlation (R = 0.92) between MCS and number of MU in this study.

### 3.B Film analysis

Calculated dose distributions from the TPS were compared with measured static and dynamic film dose distributions via profile assessment and gamma analysis.

### 3.C Profile assessment

**Fig. 3:**
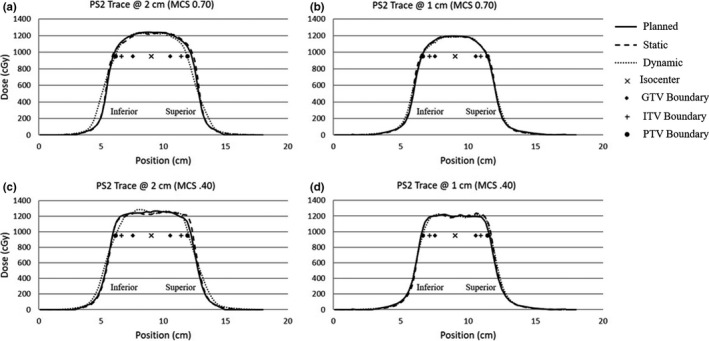
Longitudinal profiles. Longitudinal profile measurements between static, dynamic, and calculated dose distributions for several cases are shown. The plots also display the isocenter and the extent of the GTV, ITV, and PTV at the 95% prescription level (950 cGy). Figures 3a and 3b show longitudinal profile measurements for a simplified case (MCS = 0.70) at amplitudes of 2 cm and 1 cm respectively. Figures 3c and 3d show longitudinal profile measurements for the most complex case (MCS = 0.40) at amplitudes of 2 cm and 1 cm respectively.

**Fig. 4:**
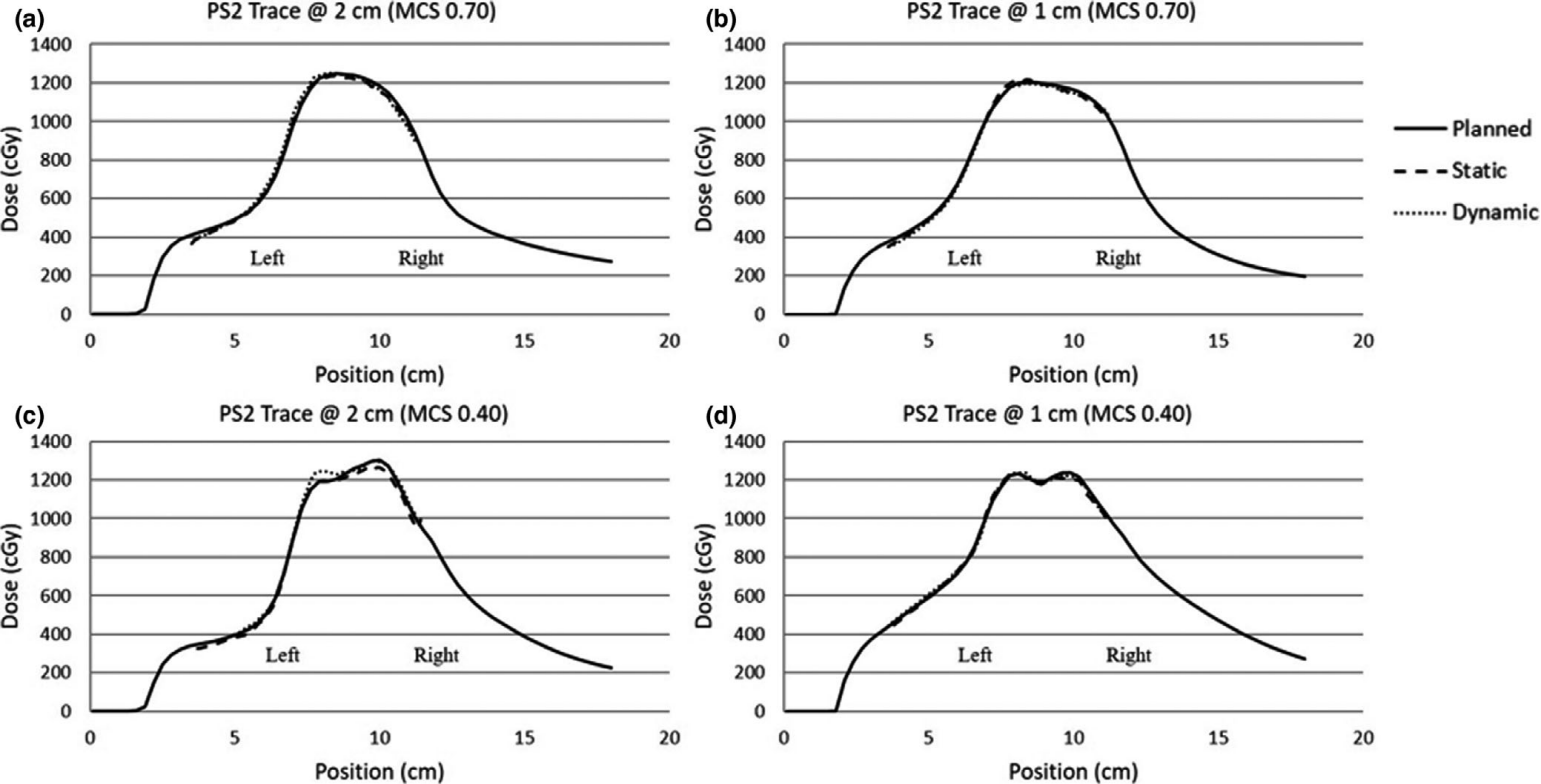
Lateral profiles. Lateral profile measurements among static, dynamic, and calculated dose distributions for several cases are shown. Figures 4a and 4b show lateral profile measurements for a simplified case (MCS = 0.70) at amplitudes of 2 cm and 1 cm respectively. Figures 4c and 4d show lateral profile measurements for the most complex case (MCS = 0.40) at amplitudes of 2 cm and 1 cm respectively.

Longitudinal and lateral profiles of each film measurement were taken. Figures 3 and 4 display profile measurements among static, dynamic, and calculated dose distributions for several cases. The plots also display the isocenter and the extent of the GTV, ITV, and PTV at the 95% prescription level (950 cGy).

Figure 3(a) shows longitudinal profile measurements for a low‐modulation case (MCS = 0.70) with an amplitude of 2 cm. The static delivery agrees with the planned dose distribution. The effects of respiratory motion on the dynamic dose distribution are visible in the edges of the profile. In the dynamic profile there was penumbra broadening and loss of coverage in the shoulder regions near the PTV. Due to the nature of the respiratory traces, as most patient spend more time in the exhale phase, the coverage in the inferior shoulder was less influenced by the respiratory motion and has more penumbra broadening. Figure 3(b) shows longitudinal profile measurements for a simplified case (MCS = 0.70) at an amplitude of 1 cm. In this plot the static and dynamic profiles agree with the planned dose distribution. The dynamic profile at 1 cm shows less penumbra broadening and less coverage loss in the shoulder of the PTV compared to the dynamic case at an amplitude of 2 cm.

Longitudinal profile measurements for a more modulated case (MCS = 0.40) with an amplitude of 2 cm are shown in Fig. 3(c). There was penumbra broadening and loss of coverage in the shoulder region. Additionally, the profiles show greater dose fluctuations in the target region for the more complex case compared to the simplified (MCS = 0.70) case. Figure 3(d) shows profile measurements for a complex case (MCS = 0.40) at an amplitude of 1 cm. There were dose fluctuations in the target region compared to the simplified case (MCS = 0.70) and there was less penumbra broadening compared to the 2 cm amplitude case.

Lateral profiles were also taken for each film delivery. Due to the design of the insert and the dose distribution in the phantom's geometry only partial lateral profiles could be obtained. Note a lateral profile is the perpendicular to the direction of phantom motion and it is seen from each measurement that there was relatively small changes due to respiratory motion between profiles for any given amplitude and complexity. Errors were most likely due to the treatment delivery, film response, film registration, or TPS model quality.

**Fig. 5:**
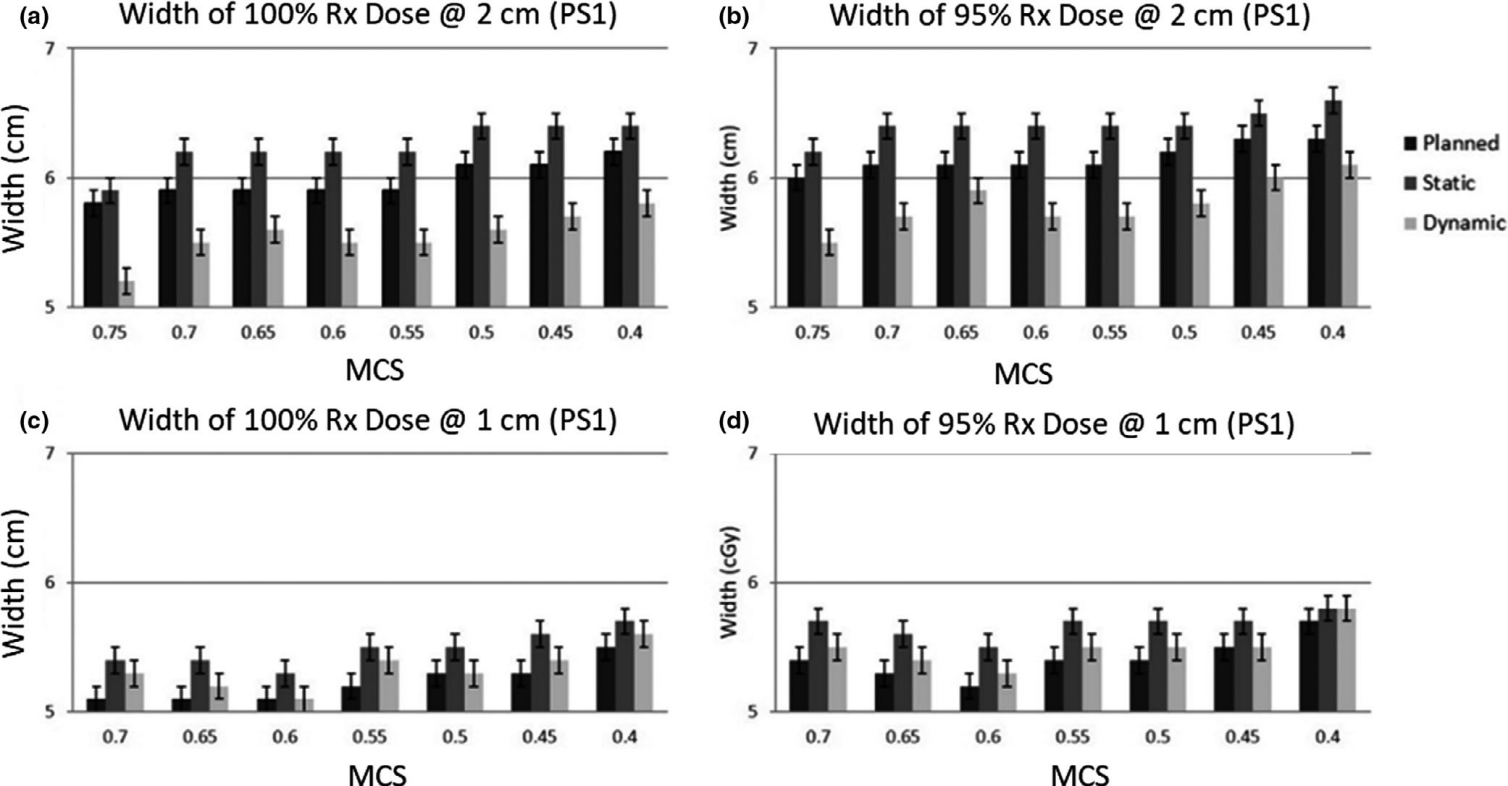
Profile Widths. The width of the 100% (1000 cGy) and 95% (950 cGy) prescription dose along the longitudinal axis for patient trace 1 are shown. In each measurement it can be seen that the width of static dose distribution is wider than the planned dose distribution. For the 2 cm deliveries, it can be seen that the dynamic dose distributions have the shortest width and that the 95% width fails to meet the required 6 cm to cover the entire PTV as prescribed.

Since the longitudinal profile corresponds to the direction of phantom motion and experiences changes in the dose distribution due to phantom motion, only the longitudinal profiles were further evaluated. The width of the 100% (1000 cGy) and 95% (950 cGy) prescription dose along the longitudinal axis for patient trace 1 are shown in Fig. 5. In each measurement it can be seen that the width of static dose distribution was wider than the planned dose distribution. For the 2 cm deliveries, it can be seen that the dynamic dose distributions had the shortest width and that the 95% width fails to meet the required 6 cm to cover the entire PTV as prescribed. However, all the 1 cm dynamic deliveries, meet the required 5 cm width to cover the entire PTV. A Pearson correlation coefficient of −0.31, 0.03, and −0.06 was calculated between the MCS and the dynamic deliveries at 2 cm, 1 cm, and all deliveries combined respectively. This indicates that there was no correlation between the MCS and the width of the distribution.

The percent dose error between the planned and delivered dose distributions at the longitudinal edges of each delivery was calculated. For the dynamic deliveries at 2 cm, we found the percent error at the superior edge (−4.06 to −22.43) was generally lower than that for the inferior edge (+6.96 to −14.02). This was influenced by the breathing trace as a patient spends more time in the end‐of‐exhale phase corresponding to the inferior edge. This trend was not evident in the dynamic deliveries at 1 cm. The percent dose error at the GTV edge was never less than 2.58% for all static and dynamic cases. The percentage dose error worsened at the ITV and PTV edges, with the measured dose being significantly lower than the calculated dose. This indicates that with sufficient margin the target coverage can be maintained. Additionally, the dynamic 2 cm irregular trace, has positive dose difference values in the inferior direction of motion for all cases, whereas the dynamic 2 cm regular traces, has negative values in the inferior direction. This was due to the nature of the irregular respiratory trace; as the irregular trace spends an abnormally large amount of time in the inferior direction.

The relative dose at the longitudinal edge of each dose distribution was calculated. Although the dose variations between planned and delivered dose distribution may seem substantial, the relative dose at the GTV edges (1137–1366 cGy) and ITV edges (1054–1263 cGy) for the dynamic deliveries were above the prescription at all amplitudes and complexity level studied. This means that the GTV and ITV coverage was satisfactorily maintained. The relative dose at the PTV edge (934–1131 cGy) for the dynamic deliveries at 1 cm, were above 90% of the prescription dose for all plans; where planning constraints suggest the PTV should not receive doses below 90%. However, the relative dose at the PTV edge (826–1163 cGy) for the dynamic 2 cm deliveries were below 90% of prescription dose for several cases.

The 2 cm dynamic deliveries show that the relative dose at the superior edge was generally lower than the inferior edge. Again, this was influenced by the breathing trace as a patient spends more time in the end‐of‐exhale phase corresponding to the inferior edge. For the 1 cm dynamic deliveries, the superior edge of the PTV for the irregular trace of the two most complex plans (MCS = 0.45 and 0.40) drop below 95% of the prescription dose. This was likely as the planned width of the 95% dose distribution for these profiles are exactly same as the required 5 cm to cover the PTV. The 2 cm amplitude dynamic irregular trace, has the largest relative dose values in the inferior direction and the lowest relative dose values in the superior direction compared to all the dynamic traces at an amplitude of 2 cm. This was due to the nature of the irregular respiratory trace; as the irregular trace spends an abnormally large amount of time in the inferior direction and consequently a small amount of time in the superior direction.

The mean, minimum, and maximum dose errors between the dynamic and planned distributions were recorded for the GTV, ITV, and PTV regions for all longitudinal profiles measurements. The results show that the mean dose difference for the GTV never dropped below 2% at any amplitude or complexity level. Also, the minimum dose in the GTV only fell below 5% for one case under irregular respirations. The minimum dose in the PTV decreases considerably for the dynamic deliveries at 2 cm. This was influenced by the loss of coverage in the shoulder of the profiles. This was determined as the magnitude of the minimum doses in the PTV does not appear in the GTV or ITV which are subsets of the PTV. Additionally, the loss of coverage in PTV shoulder appears to influence the PTV mean dose.

The width of the 100% and 95% prescription dose and the percent dose error and relative dose seen at the edges of the static dose distributions were influenced by a systematic shift seen in the film data. Looking at the dose profiles for these film measurements, a 1–2 mm shift in the longitudinal direction was observed in almost every film analyzed. The systematic shift was attributed to uncertainties in precisely identifying the isocenter within a 2.5 mm CT slice width. This uncertainty means that there is no guarantee that the isocenter defined in the TPS is at the exact same location as the actual isocenter of the phantom.

### 3.E Gamma analysis

**Fig. 6:**
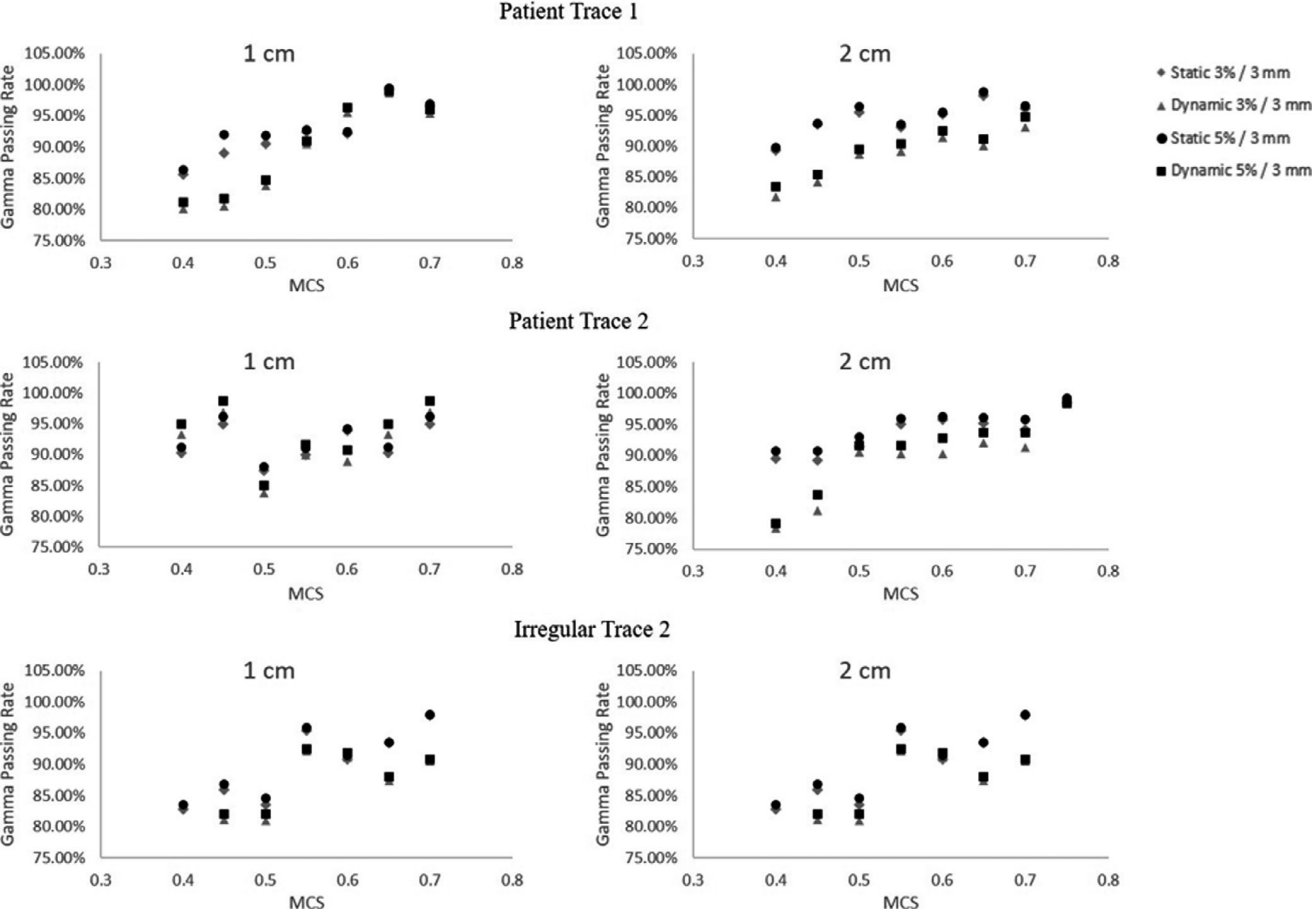
Gamma Analysis. The results show that as plan complexity increases (ie decreasing modulation complexity score) the gamma analysis passing rate decreases. Additionally, the results show that the dynamic deliveries generally have lower gamma passing rates compared to its corresponding static delivery and commonly the 2 cm dynamic deliveries have the lowest gamma passing rate.

Gamma analysis was also used to assess the agreement between planned and delivered dose. Figure 6 shows RIT's gamma analysis results. The software reports the percent of pixels passing the gamma test for the registered dose distributions. The results show that as plan complexity increases (ie decreasing modulation complexity score) the gamma analysis passing rate decreases. Additionally, the results show that the dynamic deliveries generally have lower gamma passing rates compared to its corresponding static delivery and commonly the 2 cm dynamic deliveries have the lowest gamma passing rate.

## 4 Discussion

### 4.A Summary of findings

In this study, we performed a comprehensive investigation of VMAT SBRT delivery for lung treatment. To achieve this, a respiratory motion phantom was taken through the radiotherapy imaging, planning, and delivery stages. Interplay effects on dose delivery were studied under varying degrees of plan modulation. Comparisons of planned and delivered distributions were used to gauge interplay effects on the delivered dose. Additionally, correlation between target coverage and the MCS was used to gauge its ability to indicate potentially unsafe plans. As expected, respiratory motion effects were most evident for large amplitude respirations, complex fields, and small field margins. However, under all tested conditions target coverage was maintained.

The results indicated that as plan complexity increases so does the number of MUs seen in the treatment plan (R = 0.92). This finding is inconsistent with previous work from McNiven et al.^16^ In their study the MCS and number of MU was investigated for correlation between different treatment sites; in which no correlation was found. Also, their study found limited linear correlation (R = 0.41) between the number of MU's and MCS for a variety of lungs plans. However, this study is less generalized as each plan was based on the exact same phantom geometry.

The profile assessment results show that the effects of respiratory motion are most evident for larger amplitude deliveries and at the edges of the dose distribution (ie the shoulder and penumbra). This was anticipated since previous studies have noted that the larger the amplitude the more the dosimetric deviations from the planned distribution^6^, ^10^. The dose variation between planned and delivered distribution at the edges were the largest for the most complex plans at the larger amplitudes (2 cm); where this variation reached up to 22%. However, the relative dose values indicated that the GTV, ITV, and PTV at amplitude of 1 cm maintained satisfactory coverage for the dynamic deliveries at all complexity level studied. Furthermore, the results showed that the relative dose at the PTV edge for the dynamic 2 cm deliveries were below 90% of prescription dose for several cases; where planning constraints suggest that the PTV should not receive doses below 90%.

These data were further supported by the mean, minimum, and maximum dose deviations between planned and delivered profile points. The smallest deviations were seen in the GTV. Where the mean dose never dropped below 2% and the minimum dose seen was 5.05% for the irregular patient trace at the highest degree of complexity. As you extend out to the ITV and PTV the magnitude of the dose variations increase. However the mean, minimum, and maximum data are influenced by treatment planning system modeling quality. As MLC modulation increases, the quality of the TPS model becomes important to get an accurate plan. The fluctuations seen are due to limitations in the TPS to model plans with increased modulation. This is evident as the dose fluctuations appear in the static and dynamic deliveries. Therefore, the minimum and maximum dose may be influenced by the dose fluctuations from TPS modeling quality.

A correlation coefficient of less than 0.3 was considered no correlation. The MCS did not correlate with the width, relative dose at GTV, PTV, and ITV edges, mean dose deviation, or max dose deviation for all plans. However, a limited correlation of 0.54 and 0.50 was observed for the minimum dose deviations in the ITV and PTV regions, respectively, between plan and delivered dose distributions. As expected, the MCS values correlated with the gamma analysis results for static and dynamic deliveries. The results show that as the MCS decreases, the gamma analysis results generally decreases. The correlation values were 0.85, 0.87, 0.81, and 0.57 for the mean passing rates at 5%/3 mm for the static 1 cm, dynamic 1 cm, static 2 cm, and dynamic 2 cm cases of the GTV respectively. Additionally, a correlation value of 0.65 was obtained for the mean passing rate with all GTV cases considered. This find is consistent with previous work from Masi et al.^17^ In their study the MCS displayed a positive correlation (R> 0.6) with gamma analysis results (2%/2 mm) for static VMAT deliveries at 2 Gy. Also, their study showed that as the MCS values decreased (more modulation) the gamma analysis results dropped below 90% for EBT2 film measurements. These results are influenced by MLC positioning error and the limited accuracy of MLC modeling in the TPS.

### 4.B Limitations

One limitation to this work was the Quasar respiratory motion phantom simulates 1D motion in the longitudinal direction. Although respiratory motion is usually larger in the longitudinal direction, studies have shown that lung tumors move in all three directions.^23^ Thus, it would be interesting and useful to see the effect of 3D tumor motion on the dose delivery.

As the film dosimetry process is complex, the accuracy and reproducibility of the measurement procedure could be improved. The quality of the results is directly related to this process. This includes cutting of the film pieces to fit the insert, positioning of the film in the insert, the phantom ability to accurately reproduce the respiratory traces, etc. Additionally, another type of detector besides EBT3 film can potentially be beneficial.

Another limitation is that no statistical tests were performed on the film data because the given sample size lacked sufficient statistical power. Also, this study did not account for deformation of the tumor. Previous studies have shown that in structures such as lungs, tumor, and organ deformation can occur.^24^

### 4.C Future work

In the future, one could expand the number of patient specific respiratory traces to be planned, delivered, and analyzed, in order to provide a larger sample size for statistical analysis of the data. The clinical practice of SBRT is expected to increase in use for other cancers; therefore one could also expand the range of treatment sites studied. The results can be very different due to the different planning constraints, levels of modulation, target sizes, and motion characteristics. For example, the liver is another site that has been widely treated with SBRT and has considerable amount of changes in inter‐fraction position due to respiratory motion.^24^

## Conflicts of Interest

The authors have no conflicts of interest to disclose.

## Author Contribution

Desmond Fernandez contributed substantially to the design, acquisition, analysis, interpretation of data, drafting and revising of the final version to be published. Justin Sick contributed substantially to the analysis, interpretation of data, drafting and revising of the final version to be published. Jonas Fontenot contributed substantially to the design, acquisition, analysis, interpretation of data, drafting and revising of the final version to be published.
